# The Synthesis of a Core-Shell Photocatalyst Material YF_3_:Ho^3+^@TiO_2_ and Investigation of Its Photocatalytic Properties

**DOI:** 10.3390/ma10030302

**Published:** 2017-03-16

**Authors:** Xuan Xu, Shiyu Zhou, Jun Long, Tianhu Wu, Zihong Fan

**Affiliations:** 1 Key Laboratory of Three Gorges Reservoir Region’s Eco-Environment, Ministry of Education, Chongqing University, Chongqing 400045, China; xuxuan@cqu.edu.cn (X.X.); ShirleyZhou_1223@163.com (S.Z.); longjun086@163.com (J.L.); tianhuiwu109@163.com (T.W.); 2 National Centre for International Research of Low-carbon and Green Buildings, Chongqing University, Chongqing 400045, China; 3 College of Environmental and Resources, Chongqing Technology and Business University, Chongqing 400067, China

**Keywords:** upconversion, visible light photocatalysis, core-shell structure, Ho^3+^-single-doped

## Abstract

In this paper, YF_3_:Ho^3+^@TiO_2_ core-shell nanomaterials were prepared by hydrolysis of tetra-*n*-butyl titanate (TBOT) using polyvinylpyrrolidone K-30 (PVP) as the coupling agent. Characterization methods including X-ray diffraction (XRD), transmission electron microscopy (TEM), energy-dispersive X-ray spectroscopy (EDS) under TEM, X-ray photoelectron spectroscopy (XPS), fluorescence spectrometry, ultraviolet-visible diffuse reflectance spectroscopy, and electron spin resonance (ESR) were used to characterize the properties and working mechanism of the prepared photocatalyst material. They indicated that the core phase YF_3_ nanoparticles were successfully coated with a TiO_2_ shell and the length of the composite was roughly 100 nm. The Ho^3+^ single-doped YF_3_:Ho^3+^@TiO_2_ displayed strong visible absorption peaks with wavelengths of 450, 537, and 644 nm, respectively. By selecting these three peaks as excitation wavelengths, we could observe 288 nm (^5^D_4_→^5^I_8_) ultraviolet emission, which confirmed that there was indeed an energy transfer from YF_3_:Ho^3+^ to anatase TiO_2_. In addition, this paper investigated the influences of different TBOT dosages on photocatalysis performance of the as-prepared photocatalyst material. Results showed that the YF_3_:Ho^3+^@TiO_2_ core-shell nanomaterial was an advanced visible-light-driven catalyst, which decomposed approximately 67% of rhodamine b (RhB) and 34.6% of phenol after 10 h of photocatalysis reaction. Compared with the blank experiment, the photocatalysis efficiency was significantly improved. Finally, the visible-light-responsive photocatalytic mechanism of YF_3_:Ho^3+^@TiO_2_ core-shell materials and the influencing factors of photocatalytic degradation were investigated to study the apparent kinetics, which provides a theoretical basis for improving the structural design and functions of this new type of catalytic material.

## 1. Introduction

With the development of industry, organic matter such as drugs, pesticides, surfactants, and raw chemical materials cause an increasing amount of pollutants in surface water, groundwater, sewage, and drinking water. It is even worse that most of these contaminants are complex and non-biodegradable, and therefore traditional water treatment methods cannot completely remove them. In recent years, photocatalysis has gained increasing attention due to the discovery of water splitting on a semiconductor electrode [1,2,3,4]. Over the past 20 years, photocatalysis has become a research topic of interest because of its practical applications in air and water remediation, self-cleaning, and self-sterilizing surfaces [5,6]. TiO_2_, a well-known semiconductor, has been intensively investigated during this time. Some studies showed that TiO_2_ represents one of the most promising materials for Surface Enhanced Raman Scattering (SERS), because of its high refractive index, versatile surface functionalization, synergistic coupling to plasmonic nanoparticles, cocatalysts, and so on. Thus it could be used to analyze and monitor the process of photocatalytic degradation [7,8,9]. Meanwhile, TiO_2_ with its extraordinary chemical stability, environmentally friendly, and biocompatible characteristics, has been intensively investigated as a benchmark material for many photocatalytic reactions [10,11,12,13,14]. However, the application range of TiO_2_ is still limited due to its wide band gap (3.2 eV) which requires ultraviolet irradiation for photocatalysts reaction [10,15,16]. As we all know, ultraviolet (UV) light only accounts for 5% of the total solar energy, while visible light and near-infrared (NIR) account for 45% and 48%, respectively.

Many approaches have been adopted to extend the absorption range of TiO_2_ towards the visible light region. Some researchers have made attempts to shift the band gap of TiO_2_ towards visible light by doping metals [17], rare earth metal [18], or non-metals [19,20,21], and by cationic substitutions [22]. Also, it have been reported that TiO_2_ coupling with nanoantennas could stimulate the photon-driven process and enhance the photodegradation rate because of the nanoantenna Surface Plasmon Resonance [23]. However, these methods may introduce defects such as increased recombination of photogenerated electrons and holes, and result in decreased stability, service life, and photocatalyst efficiency of TiO_2_, which is an even more serious problem [24,25].

Recently, rare-earth-doped upconversion (UC) nanophosphors have attracted a number of interests for their capability of extending the absorption range of TiO_2_. UC luminescence can switch the long-wave radiation into short-wave radiation through multiphoton mechanisms, so that low-energy light can be changed into high-energy light. UC material could convert visible light into UV light. Rare-earth doped fluorides with low photon energies and high quantum efficiency can be used as luminescent upconversion materials [26]. As a host material, fluorides such as NaYF_4_ [27,28,29], YF_3_ [30,31], and LiYF_4_ [32] have become the hotspot in the research of upconversion luminescence transformation. Fluoride has relatively lower phonon energy and high UC efficiency, which can reduce the loss of non-radiation [33,34]. Moreover, fluoride has advantages such as high chemical stabilization, high mechanical strength, and a simple preparation process. Studies have reported that the YF_3_ doped with rare-earth ions can emit UV light under visible light excitation [31,35]. With a favorable energy level structure and abundant transitions from UV to NIR region at various wavelengths, the rare-earth ion Ho^3+^ is one of the most important active ions in upconversion luminescence (UCL) applications [36,37]. Therefore in this paper, YF_3_:Ho^3+^ was selected as an intermediate matrix to absorb visible light and emit UV light which was then transferred to TiO_2,_ so that high photocatalytic reaction efficiency was realized.

The core-shell structure as a coupling model has the potential to increase luminous efficiency. A series of studies have shown that such methods can be used to improve the use ratio of solar energy. For example, NaYF_4_:Yb^3+^, Tm^3+^@TiO_2_ core-shell nanoparticles have been reported to emit UV and visible light under 980 nm excitation and perform with higher efficiencies [38,39]. It is reported that NaYF_4_ is better than YF_3_ as an upconversion nanocrystal host matrix, but it remains difficult to obtain these kinds of hierarchical nanostructures and achieve a uniform TiO_2_ coating. There were already some studies that have proven that YF_3_ could be coated with TiO_2_ successfully and stably [16,40,41]. This paper aims to prepare a YF_3_:Ho^3+^@TiO_2_ core/shell structure which is conducive for a TiO_2_ shell to absorb the UV light from UCL [42].

It has been previously reported that the YF_3_:Ho^3+^ nanoparticles can be synthesized by a facile hydrothermal method. Such YF_3_:Ho^3+^ nanoparticles exhibit good upconversion properties, which are conducive to emitting upconversion fluorescence around 288 nm under excitation at 450 nm [43]. In this work, to realize the good UC properties of YF_3_:Ho^3+^, a highly efficient photocatalyst was prepared by coating YF_3_:Ho^3+^ nanoparticles with TiO_2_. Therefore, the TiO_2_ could use visible light to improve the degradation efficiency of the catalyst. The YF_3_:Ho^3+^@TiO_2_ photocatalyst was prepared in this work by the hydrolysis of tetrabutyltitanate (TBOT) using PVP as the coupling agent. The influences of different dosages of TBOT on the materials’ morphology, size, and photocatalysis efficiency were investigated. In addition, the photocatalysis mechanism of YF_3_:Ho^3+^@TiO_2_ and the apparent kinetics of RhB degradation are discussed in details.

## 2. Experimental and Methods

### 2.1. Materials

All chemicals were used as received without further purification. Y_2_O_3_ (99.999%, Chengdu Kelong Chemical Co., Ltd., Chengdu, China), Ho_2_O_3_ (>99.9%, Shanghai TongNai Environmental Protection Co., Ltd., Shanghai, China), NaF (Chengdu Kelong Chemical Co., Ltd., Chengdu, China), ethanol (Chongqing Chuandong Chemical Group Co., Ltd., Chongqing, China), ethylenediamine tetraacetic acid (EDTA) (Chongqing Boyi Chemical Co., Ltd., Chongqing, China), acetic acid (CH_3_COOH) (Chongqing Chuandong Chemical Group Co., Ltd., Chongqing, China), and tetrabutyltitanate (TBOT) (Chengdu Kelong Chemical Co., Ltd., Chengdu, China) were of analytical grade.

### 2.2. Preparation of YF_3_:Ho^3+^@TiO_2_ Photocatalyst

In this paper, YF_3_ nanoparticles were first prepared based on Jun’s study [42,43], and then the YF_3_:Ho^3+^@TiO_2_ photocatalyst was prepared by hydrolysis of TBOT using PVP as the coupling agent based on Qin’s work. In preparation processes, TBOT (6.0 mL) was first dissolved in ethanol (30.0 mL) and CH_3_COOH (2.0 mL), and then the solution was vigorously stirred for 30 min to form precursor A; YF_3_:Ho^3+^ nanoparticles (0.02 g) were dispersed in ethanol (20.0 mL) and H_2_O (4.0 mL) to form precursor B. After that, precursor B was added dropwise into precursor A at a rate of 1 mL/min while stirring for 1 h. After standing for 24 h, the resulting nanoparticles were dried at 105 °C, and then calcined by a heating rate of 2 °C/min to 400 °C for 2 h. The experimental parameters of the TBOT dosage and the hydrolysis reaction time are shown in Table 1 below.

### 2.3. Photocatalytic Activity Measurements

In this research, RhB and phenol were used to test the photocatalytic activity of YF_3_:Ho^3+^@TiO_2_. The photocatalytic activity of YF_3_:Ho^3+^@TiO_2_ was evaluated via degradation of RhB and phenol under the irradiation of a 500 W Long arc xenon lamp with a UV cutoff filter (λ > 420 nm) under laboratory conditions using a Hitachi U-3010 UV-Vis spectrophotometer (Hitachi Corp., Tokyo, Japan).

For specific test procedures, first add 0.15 g of photocatalyst material in 500 mL of 5 mg/L solution of rhodamine B (RhB) and 500 mL of 5 mg/L solution of phenol respectively for a dark-reaction for half an hour, so as to achieve adsorption—desorption equilibrium between the pollutants and photocatalyst. Then, place the reaction system 30 cm away from the light source to be irradiated for 10 h. Take out 8 mL of the samples once every 2 h. Finally, perform centrifugation treatment for the samples and then test the absorbance of RhB at 552 nm using UV-Vis. Test the absorbance of phenol at 510 nm using 4-amino antipyrine as the chromogenic reagent under UV-Vis.

### 2.4. Characterization

The crystal structures of all prepared samples were characterized by X-ray diffraction (XRD) using a Rigaku D/Max-2500pc diffractometer (JEOL Ltd., Tokyo, Japan) with Cu Kα radiation, where the 2θ scanning angle ranged from 10° to 80°. The surface chemical environments were analyzed by X-ray photoelectron spectra (XPS) on a PHI5000 Versa Probe system (JEOL Ltd., Tokyo, Japan) with monochromatic Al Kα X-rays. Scanning electron microscopy (SEM) images were acquired with a JSM-7800F JEOL emission scanning electron microscope (Zeiss, Oberkochen, Germany). Energy dispersive X-ray (EDS) images were acquired with an EDX-100A-4 (Zeiss, Oberkochen, Germany). Transmission electron (TEM) microscopy was carried out on a FEI Tecnai G20 (JEOL Ltd., Tokyo, Japan) operated at an acceleration voltage of 200 kV. UV-Vis diffuse-reflectance spectroscopy (UV-Vis DRS) and UV-Vis absorption spectra were conducted with a Hitachi U-3010 UV-Vis spectrometer (Hitachi Ltd., Tokyo, Japan). The sample for electron spin resonance (ESR) measurement was prepared by mixing β-NaYF_4_:Ho^3+^@TiO_2_ samples in a 50 mM DMPO solution tank (aqueous dispersion for DMPO- OH and methanol dispersion for DMPO-·O_2_^−^). Upconversion photoluminescence spectra were recorded using a Horiba Jobin Yvon fluorescence spectrophotometer (Fluorolog-3; excitation source power, 0–450 W, Horiba Scientific Ltd., Tokyo, Japan).

## 3. Results and Discussion

### 3.1. X-ray Diffraction (XRD) Pattern Analysis

**Figure 1:**
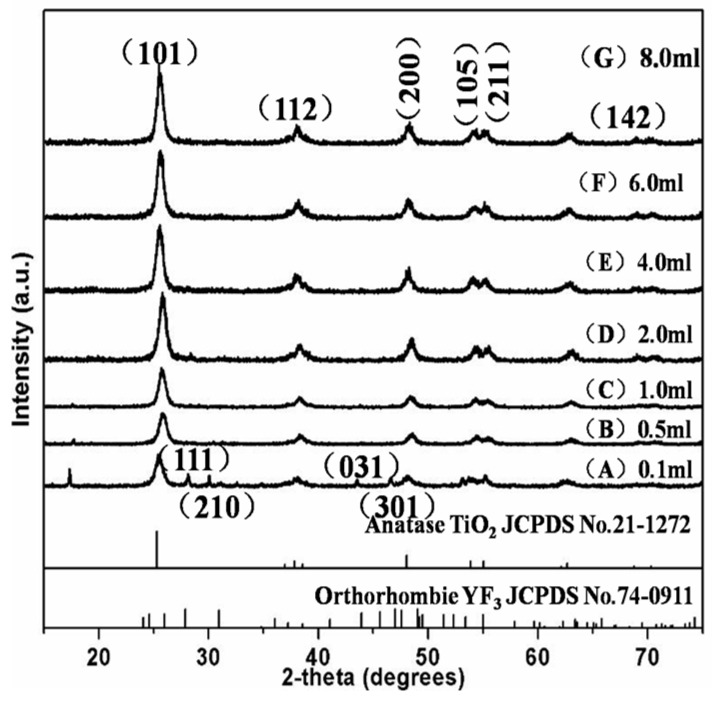
X-ray diffraction (XRD) patterns of the photocatalyst YF_3_:Ho^3+^@TiO_2_ with different tetrabutyltitanate (TBOT) dosages.

The phase structures of the materials were characterized by XRD measurements. The XRD patterns of pure TiO_2_, YF_3_, and YF_3_:Ho^3+^@TiO_2_ with different TBOT dosages are shown in Figure 1. All the diffractions of the YF_3_:Ho^3+^@TiO_2_ could be assigned to the anatase TiO_2_ (JCPDS No. 21-1272). As we all know, among all the crystal types of TiO_2_, the anatase TiO_2_ has the highest photocatalysis efficiency. Hence, the prepared materials have excellent photocatalytic capacity.

In addition, the XRD shows that only YF_3_:Ho^3+^@TiO_2_ doped with 0.1 mL TBOT coincides weakly with the YF_3_ standard (JCPDS No. 74-0911), while the others only show the diffraction peak of anatase TiO_2_. This is mainly because when the dosage of TBOT was too high, the content of TiO_2_ in the material would be relatively high, making the YF_3_:Ho^3+^ content lower than its detection limit. It may also be due to the fact that when YF_3_:Ho^3+^ was covered by TiO_2_, the strong diffraction peak of TiO_2_ would hide the diffraction peak of YF_3_:Ho^3+^, so that the diffraction peak of YF_3_:Ho^3+^ would show up only when the dosage of TBOT was reduced to a certain degree.

From the XRD results, we can see that the photocatalyst material YF_3_:Ho^3+^@TiO_2_ is synthesized by the hydrolysis of TBOT. Changing the dosage of the TBOT cannot change the phase and crystal of the UCL material, but can only affect the relative proportion between anatase TiO_2_ and YF_3_:Ho^3+^, thereby affecting the degradation efficiency.

### 3.2. Morphology and Composition Analysis by Transmission Electron Microscopy (TEM)

#### 3.2.1. TEM

**Figure 2:**
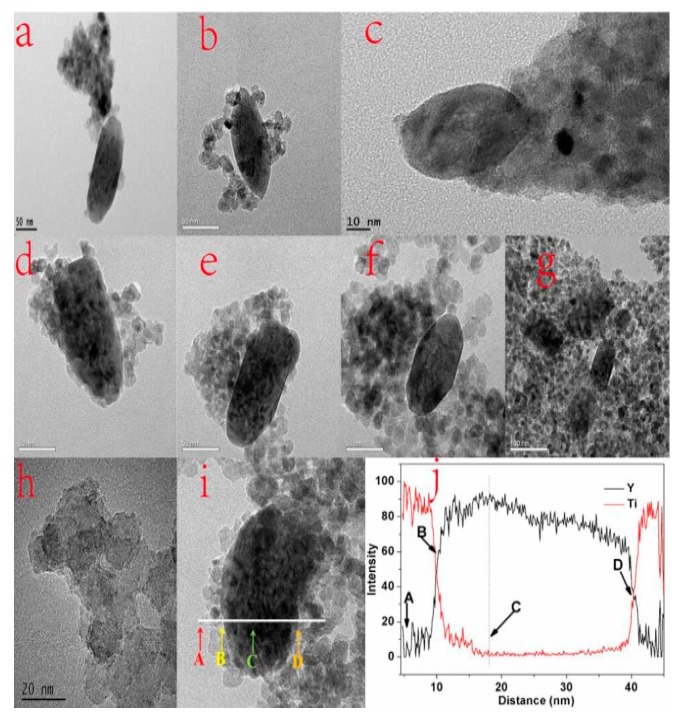
Transmission electron microscopy (TEM) images (**a**–**g**) of the YF_3_:Ho^3+^@TiO_2_ with different TBOT dosages at different magnifications; (**h**) is the corresponding high-resolution TEM image. Energy dispersive X-ray (EDS) line scan profiles and a TEM image of the YF_3_:Ho^3+^@TiO_2_ composite is shown in **i** and **j**. Points **A**–**D** in (**j**) correspond to the same point shown in (**i**).

The TEM images of YF_3_:Ho^3+^@TiO_2_ with different dosages of TBOT are shown in Figure 2a–g, from which we can see that TiO_2_ nanoparticles with particle sizes of about 10 nm stick to the UCL material. YF_3_:Ho^3+^@TiO_2_ is overall homogeneous, but in an agglomerated state. The amount of TiO_2_ doped on the YF_3_:Ho^3+^ increases with the increase of the TBOT dosage. When the dosage of TBOT is small, TiO_2_ cannot evenly coat the YF_3_:Ho^3+^, making a part of the YF_3_:Ho^3+^ still exposed. With the increase of the TBOT dosage, YF_3_:Ho^3+^ is coated by the TiO_2_ particles gradually.

#### 3.2.2. Energy Dispersive X-ray (EDS) Line Scan under High Resolution TEM (HRTEM)

Figure 2h shows the high resolution image of TiO_2_ shell coated on the surface of the YF_3_. Figure 2j shows the EDS line scanning profiles which are recorded along the white line as presented in Figure 2i. The EDX elementary line scanning was used to further determine the composition of the composite, and to prove whether the synthesized materials were of a core-shell structure or not. As shown in Figure 2j, there are signal detections of both YF_3_:Ho^3+^ and TiO_2_ at point A where the scanning starts. As the scanning goes outside and comes close to point B, the signal of Ti drops while Y increases. At point C, Ti gradually reduces to the minimum level while Y grows to the maximum level. When the scanning reaches the other end of YF_3_:Ho^3+^ at point D, Y begins to decrease while Ti gradually increases. This is obvious evidence to prove that the synthesized materials have a core-shell structure and the TiO_2_ is strongly coupled on YF_3_:Ho^3+^.

### 3.3. Chemical States Investigation by X-ray Photoelectron Spectroscopy (XPS)

**Figure 3:**
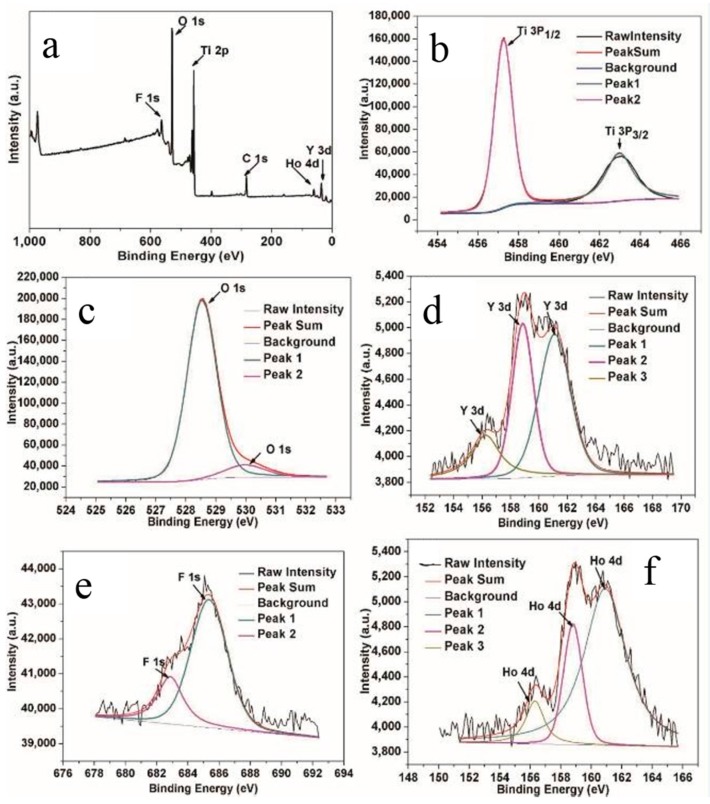
High-resolution X-ray photoelectron spectroscopy (XPS) analysis of the YF_3_:Ho^3+^@TiO_2_: (**a**) Wide spectrum; (**b**) Ti 2p; (**c**) O 1s; (**d**) Y 3d; (**e**) F 1s; (**f**) Ho 4d.

X-ray photoelectron spectroscopy (XPS) was used to examine the chemical states of the elements on the surface of the YF_3_:Ho^3+^@TiO_2_ core-shell materials. Figure 3a shows the full survey spectrum which reveals the co-presence of Ti, O, Y, F, and Ho. In Figure 3b, the binding energy of 462.98 and 457.28 eV, which are respectively labeled as Ti 2p_1/2_ and Ti 2p_3/2_, are consistent with the typical values reported for TiO_2_ [44]. According to the asymmetric profile of O 1s shown in Figure 3c, it can be seen that more than one kind of oxygen species exists. It was reported that when the binding energy was around 528.58 eV, the peak corresponded to the characteristic peak of Ti–O–Ti; while if the binding energy was around 530.08 eV, the peak was attributed to H–O. According to Figure 3d, the three peaks at 156.38, 158.88, and 161.08 eV all corresponded to Y 3d. Element F shows two peaks at 682.78 and 685.28 eV, respectively, which correspond to F 1s (see Figure 3e). In addition to the main elements in the YF_3_ nanoparticles, the doping elements in these nanoparticles were also detected. The peaks at 156.28, 158.78, and 160.88 eV (see Figure 3f) are attributed to the Ho^3+^ ions. XPS results show that rare earth ions have been successfully incorporated into the YF_3_ host matrix.

### 3.4. Optical Spectra Investigation

#### 3.4.1. UV-Vis Diffuse Reflection Spectroscopy

**Figure 4:**
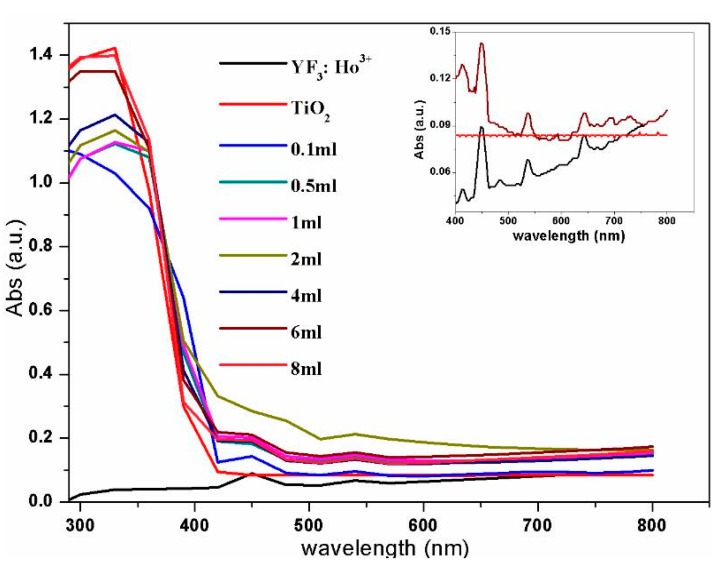
UV-Vis absorption spectra of upconversion nanoparticles (the inset shows the enlarged figure of UV-Vis absorption spectra from 400 to 800 nm).

To analyze the optimal absorption wavelength of the synthesized YF_3_:Ho^3+^@TiO_2_ material, the UV-Vis diffuse reflection spectroscopy was investigated. Figure 3 shows the representative spectra of YF_3_:Ho^3+^@TiO_2_ and YF_3_:Ho^3+^. From the spectrum of YF_3_:Ho^3+^@TiO_2,_ we can observe a light absorption edge before 400 nm, which is overlapped with that of TiO_2_. Moreover, it confirms that the YF_3_:Ho^3+^@TiO_2_ material can absorb the light with wavelengths between 300 and 700 nm, which is shown in the spectrum of YF_3_:Ho^3+^. There are three absorption peaks in the visible light region (450 nm, 538 nm, 644 nm), where the intensity of the 450 nm peak is relatively higher. As we can see from Figure 4, for one wavelength, the stronger the absorption peak’s light absorption ability is, the more suitable that wavelength is for excitation. Hence, 450 nm was selected as the excitation wavelength of YF_3_:Ho^3+^@TiO_2_, which is consistent with the goal of utilizing visible light as the excitation source for UCL materials.

#### 3.4.2. Fluorescence Spectrum Analysis

**Figure 5:**
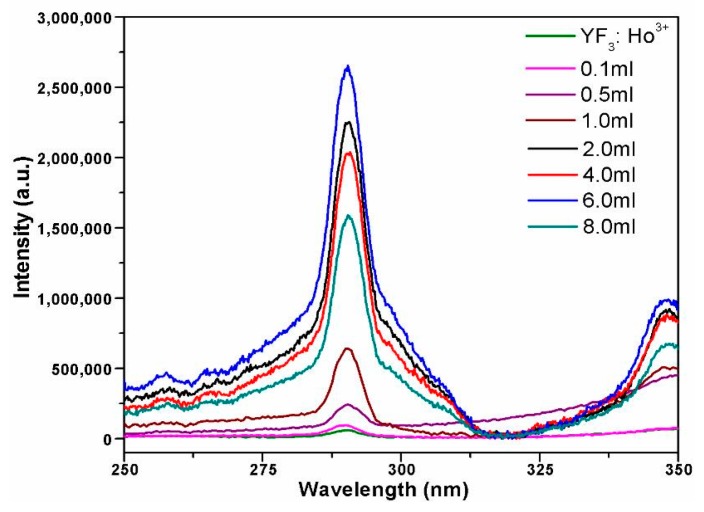
Upconversion luminescence spectra of YF_3_:Ho^3+^@TiO_2_ photocatalysts with different TBOT dosages.

Figure 5 shows the fluorescence emission spectra of YF_3_:Ho^3+^@TiO_2_ under the visible light excitation at 450 nm, from which we can see that all the prepared samples share the similar upconversion luminescence properties as YF_3_:Ho^3+^. There is a strong emission peak at 288 nm, which resulted from the transition of the Ho^3+^ ion from ^5^D_4_→^5^I_8_. When YF_3_:Ho^3+^ is doped with TiO_2_, the upconversion luminescence capacity becomes weaker. This may be due to the fact that the UV light emitted by the UCL after absorbing visible light would be absorbed by TiO_2_, and moreover, with the increase of the TBOT dosage, the TiO_2_ doped on the YF_3_:Ho^3+^ would hinder the excitation light from arriving at the YF_3_:Ho^3+^, resulting in reduced excitation light energy for YF_3_:Ho^3+^ [45]. In addition, a large number of TiO_2_ loaded on the YF_3_ surface would also absorb most of the UV light and decrease the excitation intensity of YF_3_:Ho^3+^.

### 3.5. Photocatalysis Mechanism of RhB Degradation

According to Jun’s study [43], there are two upconversion mechanisms for YF_3_:Ho^3+^, of which one is a two-photon upconversion fluorescence mechanism and the other one is a three-photon upconversion fluorescence mechanism.

To further determine the photocatalysis mechanism of YF_3_:Ho^3+^@TiO_2_, this paper detected the photogenerated radicals during the photocatalysis process by the ESR technique [46,47,48].

**Figure 6:**
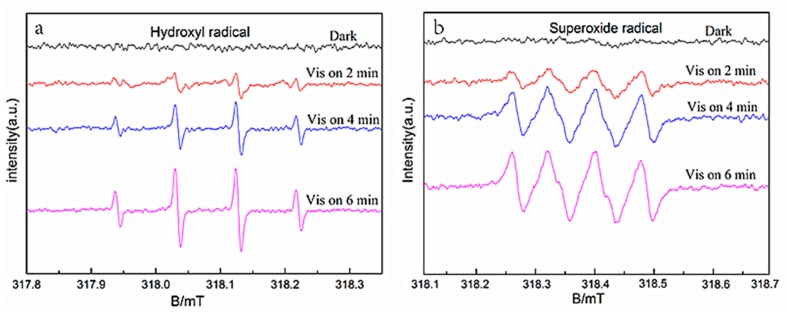
DMPO spin-trapping electron spin resonance (ESR) spectra of the YF_3_:Ho^3+^@TiO_2_ composite in the methanol dispersion for ·OH (**a**) and in the aqueous dispersion for ·O_2_^−^ (**b**).

For the capacity of generating radicals, such as the DMPO-hydroxyl radical (·OH) and DMPO-superoxide radical (·O_2_^−^), 5,5-dimethyl-1-pyrroline-*N*-oxide (DMPO) has been generally used for trapping radicals. According to the results shown in Figure 6, we can see that the signals of ·OH and ·O_2_^−^ are obvious and clear. The intensities of these two radicals’ signals increase considerably after irradiation for 6 min. Hence, the ·OH and ·O_2_^−^ are two main oxidative species for the YF_3_:Ho^3+^@TiO_2_ system. Moreover, RhB is speculated to react with these two radicals during its photocatalytic degradation, and the response equation is shown as follows.

(R1) $${\mathrm{YF}}_{3}{:\mathrm{Ho}}^{3\;+\;}\;+\;\mathrm{visible}\;\mathrm{light}\;\to {\;\mathrm{YF}}_{3}{:\mathrm{Ho}}^{3\;+\;}\;+\;\mathrm{UV}$$

(R2) $${\mathrm{TiO}}_{2}\;+\;\mathrm{UV}\;\to {\;\mathrm{TiO}}_{2\;}{\;+\;(h}^{\;+\;}{\;+\;e}^{-})$$

(R3) $$H_{2}{O\;+\;\mathrm{TiO}}_{2}\left(h^{\;+\;}\right)\;\to {\;\cdot \mathrm{OH}\;+\;H}^{\;+\;}$$

(R4) $${\cdot O}_{2}^{-}{\;+\;H}^{\;+\;}{\;+\;\mathrm{TiO}}_{2}\left(e^{-}\right)\;\to {\;\cdot O}_{2}^{-}{\;+\;H}_{2}O_{2}$$

(R5) $${\cdot O}_{2}^{-}{\;+\;H}_{2}O_{2}\;\to {\;\cdot \mathrm{OH}\;+\;\mathrm{OH}}^{-}{\;+\;O}_{2}$$

(R6) $${\cdot \mathrm{OH}(\mathrm{or}\cdot O}_{2}^{-})\;+\;\mathrm{RhB}\;\to \;\mathrm{degradation}\;+\;\mathrm{products}$$

**Figure 7:**
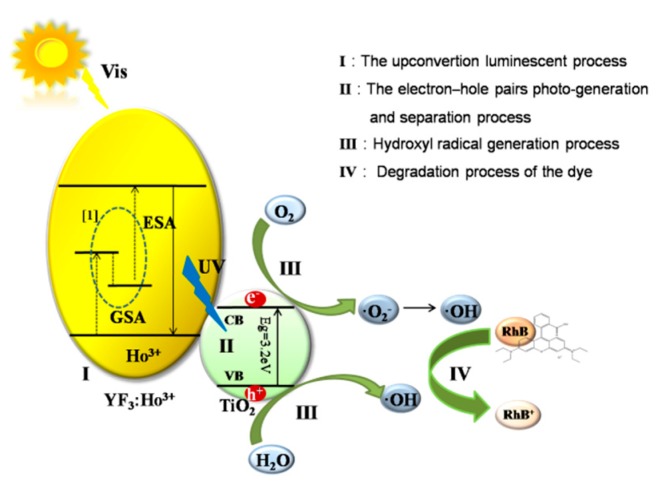
YF_3_:Ho^3+^@TiO_2_ photocatalysis mechanism for RhB degradation (the insets show the transition of Ho^3+^) Ps: [1] Xu, X. et al. Synthesis and intense ultraviolet to visible upconversion luminescence of YF^3^:Ho^3+^ nanoparticles. GSA: ground state absorption; ESA: excitation state absorption; UV: ultraviolet; VB: valence band; CB: conduction band.

Besides, based on the response equation, this paper also theorizes a possible reaction process shown in Figure 7. Firstly, the UCL material YF_3_:Ho^3+^ emits UV light after absorbing visible light (R1). Then, TiO_2_ trapped on the UCL surface will generate electron-hole pairs through UV excitation (R2). The excited electrons on the TiO_2_ surface react with oxygen to form ·O_2_^−^ and HO_2_, and then the resulted HO_2_ combines with H^+^ to form hydrogen peroxide later (H_2_O_2_; R4). When ·O_2_^−^ meets H_2_O_2_, it will generate ·OH (R5); meanwhile, the photogenerated holes at (R2) will react with H_2_O to form ·OH (R3). For the conduction band of TiO_2_ located above the RhB redox potential, the oxygen species (·OH, ·O_2_^−^, and H_2_O_2_) could oxidize the RhB to realize the purpose of RhB’s degradation.

### 3.6. Photocatalysis Application

**Figure 8:**
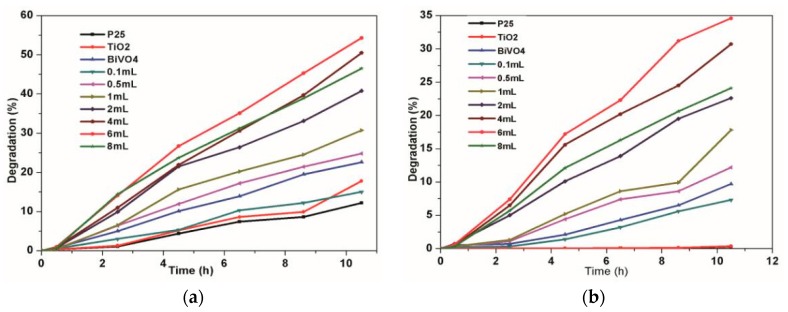
Effect of TBOT dosage on the removal rate of RhB (**a**) and phenol (**b**).

Figure 8a,b show the influences of YF_3_:Ho^3+^@TiO_2_ with different dosages of TBOT on RhB and phenol degradation, respectively. All the samples have almost zero adsorption efficiency both for RhB and phenol, wherein the most significant one is less than 1%. For the RhB and phenol, the photocatalytic degradation efficiency first increases then decreases with the increase of the TBOT dosage. When the dosages of TBOT are 6 mL, it can obtain the highest degradation efficiency (67%) of RhB as well as the highest degradation efficiency (34.6%) of phenol. According to the analyses above, this could be due to the fact that when the TBOT dosage increased, more TiO_2_ would be doped on the YF_3_:Ho^3+^, and thus more UV light would be absorbed, resulting in more excited electron-hole pairs and an improved degradation efficiency of RhB and phenol. However, when the dosage of TBOT continued to increase to 8 mL, the degradation efficiency would be decreased for both RhB and phenol. According to the TEM of YF_3_:Ho^3+^ in Figure 3, when the dosage reaches 8 mL, the YF_3_:Ho^3+^ is covered by TiO_2_ completely, which will affect the absorption of visible light and the conversion from visible light to UV light, thus reducing the photocatalytic activity of YF_3_:Ho^3+^@TiO_2_.

This paper also tested the photocatalytic efficiency of P25, TiO_2_ (prepared using a similar method as YF_3_:Ho^3+^@TiO_2_ without YF_3_:Ho^3+^), and BiVO_4_, respectively. For RhB degradation, the results showed that the efficiency of P25 was 12.2%, the efficiency of TiO_2_ was 17.8%, and the efficiency of BiVO_4_ was 22.6%. For phenol degradation, the results showed the efficiency of P25 and TiO_2_ was nearly zero, and the efficiency of BiVO_4_ was 9.7%, all of which were less than that of YF_3_:Ho^3+^@TiO_2_. Therefore, it can be concluded that the YF_3_:Ho^3+^@TiO_2_ material can make up for the defects of TiO_2_ (which is unable to have a photocatalytic reaction under visible light irritation), and has a higher photocatalytic efficiency than the common visible light photocatalyst BiVO_4_.

### 3.7. Influencing Factors of the Photocatalytic Degradation Reaction

The influencing factors of photocatalytic reaction include photocatalyst dosage (*m*_cata_), substrate concentration (*C*_0_), and irradiation intensity (*E*), which are also the three major factors that affect the *k*_a_ coefficient of the photocatalytic kinetics equation, according to the Langmuir-Hinshelwood equation (other factors are not considered). This paper has studied a series of experiments to figure out how the apparent photocatalytic degradation kinetics change with these three factors.

#### 3.7.1. The effect of *m*_cata_

**Figure 9:**
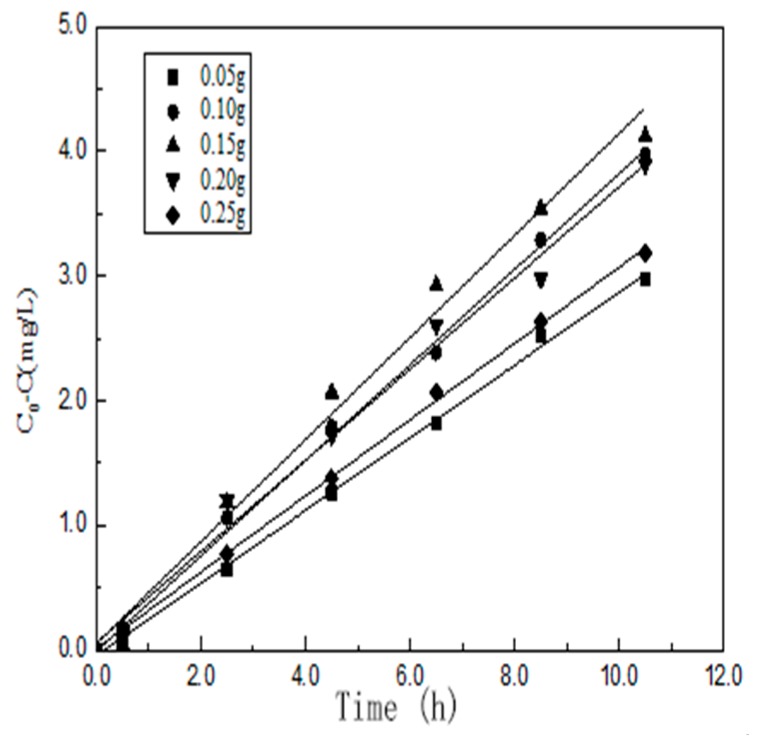
Degradation rate of RhB with different dosages of the photocatalyst.

Figure 9 shows how different dosages of YF_3_:Ho^3+^@TiO_2_ affect the photocatalytic efficiency. For the experiment, this paper changes the dosages of the photocatalyst from 0.05 to 0.25 g. And the result shows that with the increasing YF_3_:Ho^3+^@TiO_2_ dosage, the degradation efficiency of RhB first increases and then decreases. This may be due to the fact that the increased photocatalyst dosage not only improved the photon efficiency to generate more photogenerated radicals, but also increased effluent turbidity, causing light scattering to decrease photon efficiency. After fitting, an equation can be obtained as shown below.

$$k_{a}\;=\;-10{.643\;m}^{2}\;+\;3.2156\;m\;+\;0{.161\;(R}^{2}\;=\;0.9717)$$

If${\;k}_{a}{\;=\;k}_{1}m^{b}$, i.e., ${\ln k}_{a}{\;=\;\mathrm{lnk}}_{1}\;+\;\mathrm{bln}m$, there will be two conditions, *m* = 0.05 g–0.15 g and *m* = 0.15 g–0.25 g. Through the ln*k*-ln*m* graph, we can obtain k_1_ = 0.76638, b = 0.31835 (R^2^ = 0.93077) and k_1_ = 0.14273, b = −0.56254 (R^2^ = 0.92079). Then there is:

$$k_{a}\;=\;0.76638\;m^{0.31835}\;\left(0.05\;\leq \;m\;\leq \;0.15\right)$$

$$k_{a}\;=\;0{.14273\;m}^{-0.56254}\;(0.15\;\leq \;m\;\leq \;0.25)$$

#### 3.7.2. The effect of *C*_0_

This paper tested different concentrations of substrate (RhB) for photodegradation.

**Figure 10:**
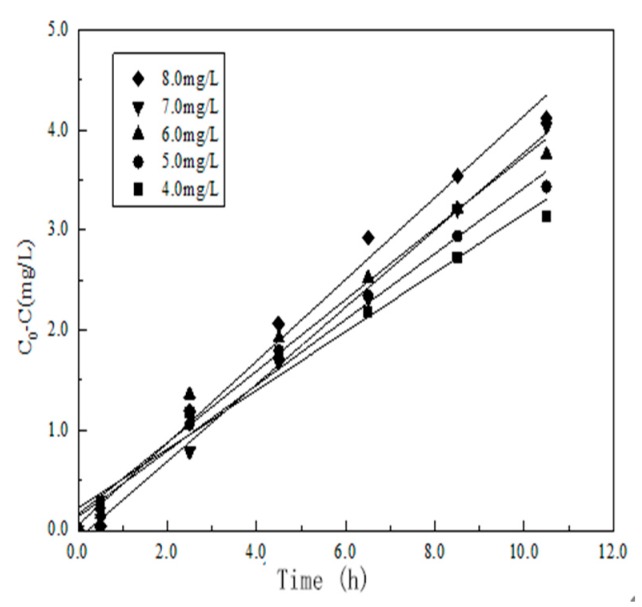
Degradation rate of RhB with different concentrations of initial glucose.

Figure 10 shows how different concentrations of substrate affect the photocatalytic efficiency. The degradation efficiency of RhB decreases with the increase of the substrate concentration. When the concentration of the substrate is 4 mg/L, the photocatalytic efficiency reaches the highest value, which is 76%, and when it is 8 mg/L, the photocatalytic efficiency reaches the lowest value, which is 56%. This is mainly because when the concentration of the substrate is relatively low, the photocatalyst YF_3_:Ho^3+^@TiO_2_ is in excess, and then the photocatalytic efficiency of RhB will be relatively high. On the contrary, when the concentration of the substrate is high, the photocatalyst YF_3_:Ho^3+^@TiO_2_ is no longer sufficient, and all of the photocatalyst needs to take part in the photodegradation. At this time, while the photodegradation rate is at its highest value, the apparent degradation efficiency will decrease with the increase of the substrate concentration, and thus photodegradation efficiency will be worse. However, when the concentration of the substrate is too high, it will reduce the light transmittance of the solution, thus reducing the photocatalytic activity.

After fitting, an equation can be obtained as shown below.

$$k_{a}\;=\;0{.0287C}_{0}\;+\;0{.18279\;(R}^{2}\;=\;0.99324)$$

#### 3.7.3. The effect of irradiation intensity

**Figure 11:**
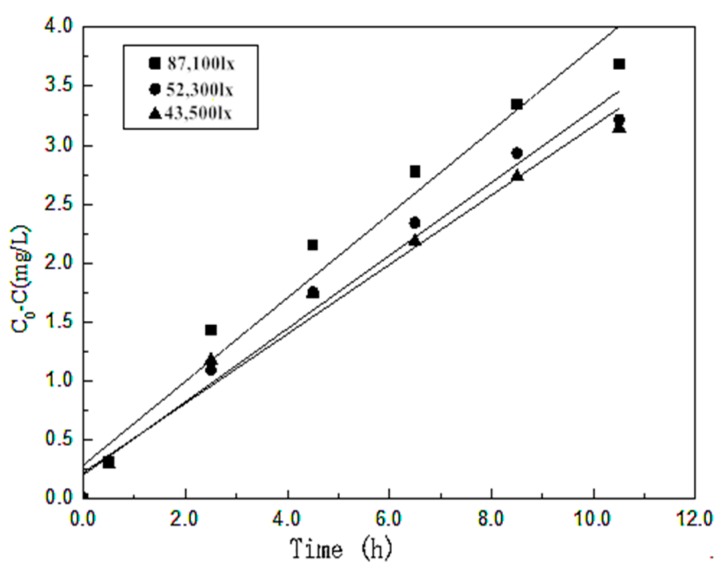
Degradation rate of RhB with different irradiation intensities.

Figure 11 shows how different irradiation intensities affect the photocatalytic efficiency. When the irradiation intensity reaches 141,500 lx, each factor is at its best level, the RhB is degraded completely, and the process is not a zero order reaction. Therefore, in this paper, only the irradiation intensities of 87,100 lx, 52,300 lx, 43,500 lx were considered. With the increase of irradiation intensity, reactions between the photocatalyst and photon will increase and the rate of the photocatalysis reaction will be faster, thus leading to an increased efficiency. After fitting, an equation can be obtained as shown below.

$$k_{a}\;=\;1{.4154\;\times \;10}^{-6}E\;+\;0{.2318\;(R}^{2}\;=\;0.97489)$$

The apparent kinetics model of RhB degradation is

$$C_{A}{\;=\;C}_{0}\;-\;2{.19967\;\times \;10}^{-6}m^{0.31835}C_{0}Et$$

(0.05 g ≤ *m* ≤ 0.15 g, 4.0 mg/L ≤ *C*_0_ ≤ 8.0 mg/L, 43500lx ≤ *E* ≤ 87100lx)

$$C_{A}{\;=\;C}_{0}\;-\;4{.97\;\times \;10}^{-7}m^{-0.56254}C_{0}Et$$

(0.15 g ≤ *m* ≤ 0.25 g, 4.0 mg/L ≤ *C*_0_ ≤ 8.0 mg/L, 43500lx ≤ *E* ≤ 87100lx)

## 4. Conclusions

To solve the problem of TiO_2_ having nearly no photocatalytic efficiency under visible light irradiation, a composite photocatalyst material YF_3_:Ho^3+^@TiO_2_ was prepared in this paper using upconversion luminescence technology. Through analyzing the morphology and composition, crystal structure, and optical spectra of YF_3_:Ho^3+^@TiO_2_, it was found that this material had high photocatalytic efficiency under visible light irradiation. In addition, this paper also investigated the influences of different dosages of TBOT on the properties of the photocatalyst.

In summary, YF_3_:Ho^3+^@TiO_2_ can be successfully prepared using a simple hydrothermal method. By analyzing the XRD images, we found that almost all the samples showed the diffraction peak of anatase TiO_2_ and that the crystal structure of the material did not change with TBOT dosage. TiO_2_ was uniformly doped on UCL, and the particle size was about 10 nm. The rice-shaped UCL material had good dispersion, of which the particle size was about 100 nm. The change of TBOT dosage would not cause the change of the material morphology, but would cause the change of the amount of TiO_2_ doped on UCL, resulting in an impact on the photocatalytic activity of YF_3_:Ho^3+^@TiO_2_. The composite material prepared in this paper shared the same upconversion luminescence property with the UCL material prepared by Jun. It confirmed that the material prepared in this paper could absorb 450 nm visible light and emit UV light, with energy transferred from YF_3_:Ho^3+^ to anatase TiO_2_. With the increase of the TBOT dosage, more TiO_2_ would be doped on the YF_3_:Ho^3+^, therefore the excitation light was obstructed, resulting in a lower energy of exciting light. The photocatalytic properties of the YF_3_:Ho^3+^@TiO_2_ was evaluated by the degradation of RhB and compared with those of traditional photocatalysts such as P25, TiO_2_, and the visible light photocatalyst BiVO_4_. The results showed that the prepared composite material exhibited better photocatalytic properties as compared with the other three photocatalysts. With the increase of the TBOT dosage, the photocatalytic efficiency of composite YF_3_:Ho^3+^@TiO_2_ first increased and then decreased. When the TBOT dosage was 6 mL, the photocatalytic efficiency reached the highest value, which was 67%. The results of this paper indicated that Ho^3+^-single-doped hexagonal YF_3_ could absorb visible light and emit UV light via UC processes. Under UV light irradiation, the composite material YF_3_:Ho^3+^@TiO_2_ could exhibit better photocatalytic properties than that of anatase TiO_2_, therefore the prepared composite material YF_3_:Ho^3+^@TiO_2_ has promising applications in photocatalysis.

## Figures and Tables

**Table 1:** Synthesis condition of all samples. TBOT: tetrabutyltitanate.

Number	Dosage of TBOT/mL	Hydrolysis Reaction Time/min
A	0.1	60
B	0.5	60
C	1	60
D	2	60
E	4	60
F	6	60
G	8	60
